# Advances in Hemophagocytic Lymphohistiocytosis: Pathogenesis, Early Diagnosis/Differential Diagnosis, and Treatment

**DOI:** 10.1100/tsw.2011.62

**Published:** 2011-03-22

**Authors:** Yong-Min Tang, Xiao-Jun Xu

**Affiliations:** Division of Hematology-oncology, Children's Hospital of Zhejiang University School of Medicine, Hangzhou, China

**Keywords:** hemophagocytic lymphohistiocytosis, genetics, biomarker, Th1/Th2 cytokine pattern

## Abstract

Hemophagocytic lymphohistiocytosis (HLH) is a histiocytic disorder characterized by a highly stimulated, but ineffective, immune response to antigens, which results in life-threatening cytokine storm and inflammatory reaction. Considerable progress has been made during the past 2 decades. Detection of molecular genetic abnormalities in genes involved in immune response pathways, such as PRF1, STX11, UNC13D, STXBP2, RAB27A, LYST, AP3B1, SH2D1A, and BIRC4, is confirmatory for the diagnosis. Clinical diagnosis is largely made according to HLH-2004 criteria. However, a new finding of the Th1/Th2 cytokine pattern (significant increase of IFN-γ and IL-10 with slightly increased or normal level of IL-6) is a useful biomarker for the early diagnosis, differential diagnosis, and the monitoring of the disease. Intensive immunosuppressive therapy is generally accepted as treatment for the relief of clinical symptoms/signs, while allogeneic hematopoietic stem cell transplantation is currently the only potentially curative therapy option for severe familial forms of HLH.

## INTRODUCTION

Hemophagocytic lymphohistiocytosis (HLH) is a histiocytic disorder characterized by a highly stimulated, but ineffective, immune response to antigens, which results in life-threatening cytokine storm and inflammatory reaction. HLH is not a single entity, but a clinical syndrome that can be encountered in association with various underlying diseases leading to similar characteristic clinical and laboratory presentations. Causative mechanisms include underlying genetic defects as well as "trigger" factors, such as infections, malignancies, and autoimmune disorders. The elucidation of molecular mechanisms of HLH has provided important information for the understanding of its pathophysiology and has contributed greatly to the successful treatment for the majority of patients.

## GENETIC DISORDERS OF HLH

The granule-dependent cytotoxic pathway of natural killer (NK) cells and cytotoxic T lymphocytes (CTLs) is the key machinery in the clearance of viral and intracellular bacterial infections, as well as in the prevention of tumor development. Once NK cells or CTLs recognize their targets and form an immune synapse (IS), cytotoxic granules are delivered in a multistep process. This includes cytotoxic granule biogenesis and maturation, trafficking to the IS, docking, priming, and fusing with the plasma membrane so that release of their content induces target cell destruction by apoptosis[1,2] (Fig. 1). A pathological finding of HLH is a deficiency of cytotoxic function in NK cells and CTLs, without modifying their activation capacity or cytokine secretion, which occurs at any step in the process. Several genetic loci related to this activity have been identified in the pathophysiology of genetic HLH[3,4] (Fig. 1). Mutations in the perforin gene were the first to be described in familial hemophagocytic lymphohistiocytosis (FHL) patients and their families in 1999 by a group from Paris[5], which accounts for about 15–50% of FHL cases[6]. Over 70 different recessive mutations in PRF1 have now been found in FHL2 patients[7]. PRF1 mutations can result in either complete or partial reduction of perforin protein expression, a mutated protein which lacks cytotoxic function, or partial impairment of expression and function both[2,8,9,10,11,12]. The second identified cause of FHL (FHL3) is mutations in UNC13D that encodes the Munc13-4 protein[13]. Munc13-4 is required to prime cytotoxic granule membrane fusion with the plasma membrane at the IS. This step is essential for the secretion of cytotoxic granules[13,14,15]. Moreover, at an earlier step, Munc13-4 mediates the combination of late endosomes and recycling endosomes to form exocytic vesicles[15,16]. Another two variants of FHL involve syntaxin 11 and Munc18-2, which are encoded by STX11 and STXBP2, and are responsible for FHL4 and FHL5, respectively[17,18,19,20]. Syntaxin 11 is a member of the family of SNARE (soluble N-ethylmaleimide sensitive factor accessory protein receptor), which interacts with accessory proteins, such as the Munc18 family, and induces SNARE-mediated membrane fusion[21]. Munc18-2 is involved in the regulation of the assembly and disassembly of SNARE complexes and, thus, the control of membrane fusion[22]. When granules dock at the membrane, they are primed for fusion by Munc13-4, which probably triggers the switch of the Munc18-2/syntaxin 11 complex from a closed to an open conformation. A SNARE complex then forms between the vesicle membrane and the target cell membrane[1,19,23,24].

Besides the above genetic abnormalities, HLH can also occur with significant frequency in five other genetic diseases that have been linked with defective cytotoxic function in NK cells and CTLs: Griscelli syndrome (GS2)[25], Chediak-Higashi syndrome (CHS)[26], Hermansky-Pudlak syndrome type II (HPS II)[27], and X-linked lymphoproliferative syndrome type 1 (XLP1) and type 2 (XLP2)[28,29], which are characterized by mutations in the RAB27A, LYST, AP3B1, SH2D1A, and BIRC4 genes, respectively. Rab27a mediates tethering of cytotoxic granules to the membrane and then granules can dock at it[30,31]. In GS2 patients, polarized cytotoxic granules fail to dock at the plasma membrane, although the polarization is normal[32]. LYST is either involved in vesicle fusion and fission, or in the sorting of lysosomes, which is essential in cytotoxic granule biogenesis[33,34,35]. In CHS patients, cytotoxic granules are enlarged and fail to release their content at the IS, despite a normal polarization upon activation[26]. AP3B1 is the β-chain of the adapter protein-3 (AP3) complex, which regulates the transport of lysosomal proteins and is required for protein sorting to lysosomes[36,37]. In HPS II patients, the cytotoxic granules in AP3B1-deficient CTLs are incapable of moving along microtubules towards their destination[38]. XLP is caused by mutations in SH2D1A encoding signaling lymphocytic activation molecule (SLAM)–associated protein (SAP)[28]. Recent data have highlighted a role for SAP in the development, differentiation, and effector function of B cells, CD4+ T cells, CD8+ T cells, NK cells, and NKT cells[39]. Another genetic abnormality causing XLP involves the BIRC4 gene, which encodes for the X-linked inhibitor-of-apoptosis (XIAP) and has been recently described[29].

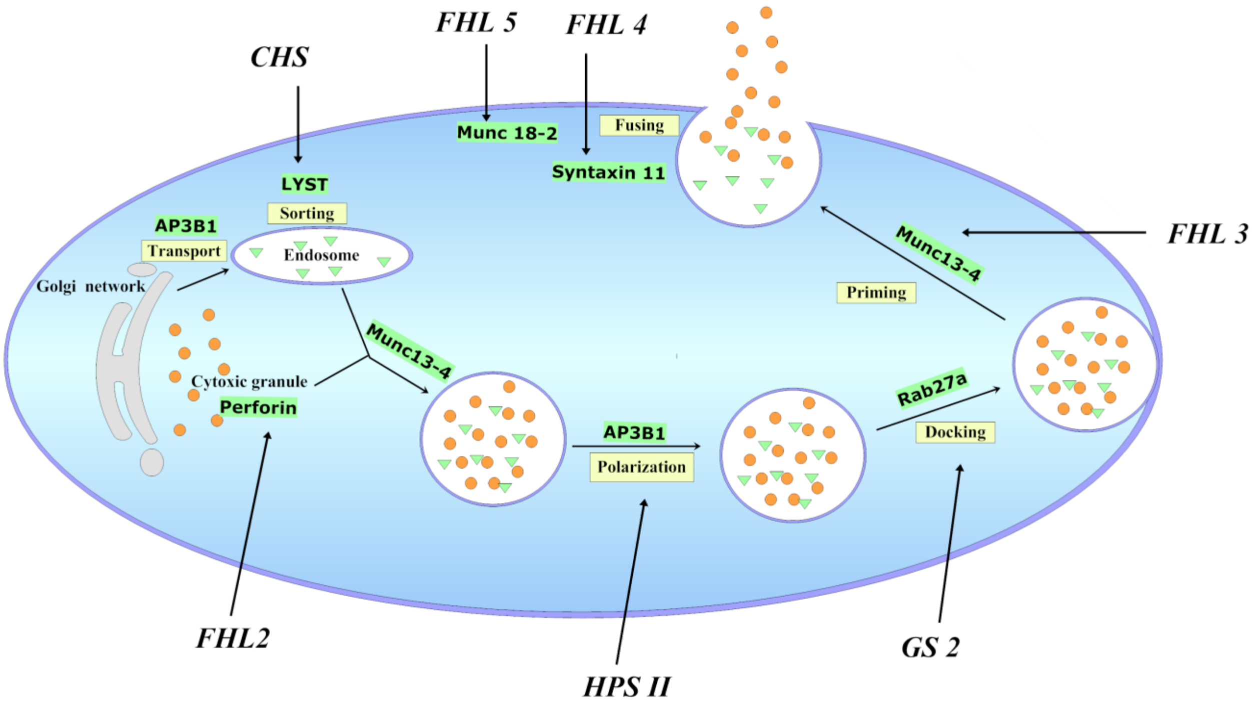

**FIGURE 1.** The schematic diagram of HLHs and their defective genes. Following T-cell receptor (TCR) stimulation, granzymes and other proteins are secreted from the Golgi network, transported to and sorted in endosomes, which then coalesce with perforin-containing granules to form mature cytotoxic granules. The granules then polarize to the IS and dock at the plasma membrane through the Rab27a. Docked granules are then primed by Munc13-4, which triggers SNARE-mediated membrane fusion. The membrane fusion is exerted by syntaxin 11 and Munc18-2. Impairment of any step in the above process may result in the deficiency of cytotoxic function in NK cells and CTLs and cause HLH.

## PATHOPHYSIOLOGY

HLH is a highly stimulated, yet ineffective, multisystem inflammatory response to antigens that is attributed to a defect of perforin-mediated cytotoxic activity of CD8$^+$T cells or NK cells (Fig. 2). The key pathophysiology of this disease is hyperactivation and hyperproliferation of CTLs and macrophages, as well as hypercytokinemia. As the ability to fully eliminate antigens fails owing to the granule-dependent cytotoxic pathway defect, persistent stimuli of antigens drive excessive, uncontrolled, and fatal antigen-specific T-cell expansion[40]. Meanwhile, perforin down-regulation on the immune response by perforin-dependent activation-induced cell death is impaired as well[41,42], resulting in CD8$^+$ T cells continuously receiving activation and proliferation signals. In this circumstance, T cells continue to proliferate and to produce a great quantity of IFN-γ, stimulating uncontrolled macrophage activation that, in turn, causes secretion of high levels of IL-12 and other cytokines, such as IL-1, IL-6, IL-10, IL-18, and TNF-α. IL-12 functions to stimulate CTLs to expand and to produce IFN-γ, and to promote the differentiation of naïve CD4$^+$ T cells into IFN-γ–producing Th1 cells. IL-18 acts synergistically with IL-12 to promote IFN-γ synthesis and T-cell activation. TNF-α and IL-1 act to recruit neutrophils and monocytes to the sites of infection, and to activate these cells to eradicate microbes. These cytokines lead to further activation, proliferation, and recruitment of additional lymphocytes and inflammatory cells. Although IL-10 inhibits Th1 cells and the production of Th1 cytokines, and negatively regulates the activated macrophages and the production of IL-12, it plays a limited role in the cytokine storm. The result is the accumulation of lymphohistiocytic infiltrates into organs, including the liver, spleen, lymph nodes, bone marrow, and central nervous system, with associated organ damage as well as the systemic signs of hypercytokinemia.

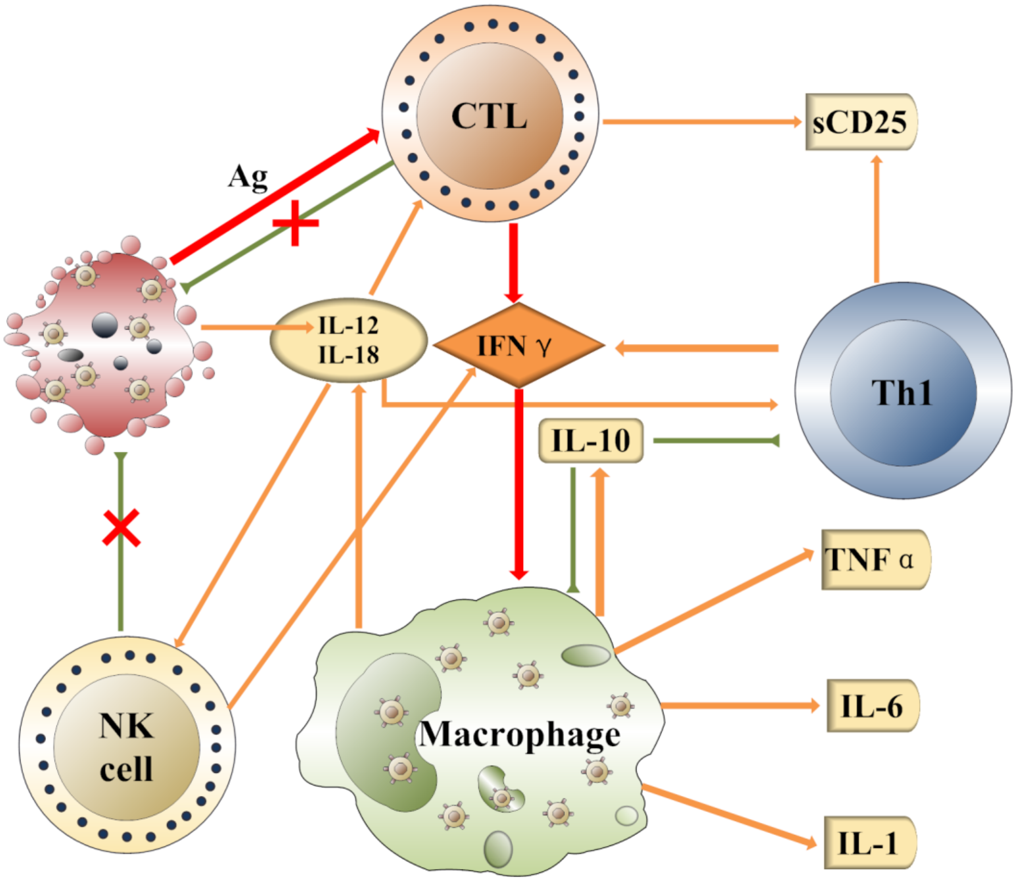

**FIGURE 2.** The proposed network of cytokine regulation associated with the pathophysiology of HLH. Pathogenic viral infection in a normal individual leads to the production of proinflammatory factors, such as IL-12, by the infected cells. This induces relevant responses from NK cells and lymphocytes. Once induced, NK cell and CTL cytotoxicity can help to rapidly eliminate the infected cells and serves to prevent further immune-cell activation. In HLH patients, the NK cells and CTLs fail to lyse the infected cells. Thus, the inflammatory response continues unabated. T cells continue to proliferate and to produce a great quantity of IFN-γ, stimulating the macrophage uncontrolled activation in turn, secreting high levels of IL-12 and other cytokines, such as IL-1, IL-6, IL-10, IL-18, and TNF-α. IL-12 functions to stimulate CTLs to expand and to produce IFN-γ. The hypercytokinemia leads to the accumulation of lymphohistiocytic infiltrates into organs and organ damage.

It has been reported that patients with HLH have high levels of various cytokines, such as IFN-γ, IL-6, IL-10, IL-12, IL-18, TNF-α, macrophage inflammatory protein (MIP1-α), and sCD25[43,44,45,46]. Activated macrophages may nonselectively engulf hematopoietic components, such as erythrocytes, leukocytes, platelets, their precursors, and cellular fragments, leading to the hallmark findings in the bone marrow or other organs of patients with HLH. High levels of cytokines and organ infiltration are associated with various signs and symptoms of HLH. Prolonged fever is caused by high levels of IL-1, IL-6, and TNF-α; cytopenia is attributed to high concentrations of TNF-α and INF-γ, and hemophagocytosis; hypertriglyceridemia resulted from increased levels of TNF-α, leading to decreased activity of lipoprotein lipase; hypofibrinogenemia may be the result of increased plasminogen activator expressed by activated macrophages that will, in turn, lead to increased serum levels of plasmin. A high level of ferritin is secreted by activated macrophages. High concentrations of sCD25 are produced by activated lymphocytes. Hepatosplenomegaly, liver dysfunction, and neurological signs and symptoms can be explained by organ infiltration with lymphocytes and histiocytes, or the direct cytotoxicity of the extremely high levels of cytokines to the tissues of the organ involved before infiltration of lymphocytes and histiocytes.

## NEW BIOMARKERS AND EARLY DIAGNOSIS OF HLH

Although FHL was first described in 1952 (termed as "familial haemophagocytic reticulosis" by Farquhar and Claireaux)[47], a set of diagnostic guidelines was first presented by the Histiocyte Society in 1991[48] and was revised in 2004[49] (Table 1). Diagnosis of HLH relies on common clinical, laboratory, and histopathological findings. Prolonged fever, hepatosplenomegaly, and cytopenias are the cardinal symptoms of HLH. Lymphadenopathy, rash, or neurological symptoms, such as seizures or cranial nerve palsies, are less frequent. Characteristic laboratory findings include high levels of triglycerides, ferritin, transaminases, bilirubin, and low fibrinogen. Hemophagocytic cells may be absent in an initial biopsy specimen, but are usually found during disease progress. Two highly diagnostic parameters are the increased plasma concentration of sCD25 and the impaired NK-cell activity. The elevation of sCD25 in HLH suggests the activation of T lymphocytes, and the impairment or absence of NK-cell activity is a hallmark of this disease.

**TABLE 1**
**Diagnostic Criteria for HLH**

| |
|---|
| The diagnosis of HLH can be established if one of either A or B criteria below is fulfilled. |
| A. A molecular diagnosis consistent with HLH |
| B. Diagnostic criteria for HLH fulfilled (five of the eight criteria below) |
| 1. Fever |
| 2. Splenomegaly |
| 3. Cytopenia (affecting ≥2 of 3 lineages in the peripheral blood): |
| - Hemoglobin <90 g/l (in infants <4 weeks: hemoglobin <100 g/l) |
| - Platelets <100 × $10^9$/l |
| - Neutrophils <1.0 × $10^9$/l |
| 4. Hypertriglyceridemia and/or hypofibrinogenemia: |
| - Fasting triglycerides ≥3.0 mmol/l (i.e., ≥265 mg/dl) |
| - Fibrinogen <1.5 g/dl |
| 5. Hemophagocytosis in bone marrow or spleen or lymph nodes without evidence of malignancy |
| 6. Low or absent NK-cell activity |
| 7. Ferritin ≥500 µg/l |
| 8. Soluble CD25 (i.e., IL-2 receptor) ≥2400 U/ml |

A retrospective report of 65 patients revealed that the median time from onset of symptoms or signs of disease to diagnosis of HLH was 3.5 months. Fever and splenomegaly are present in about three-fourths of the patients at initial presentation, while bicytopenia, hypertriglyceridemia, and ferritin >500 ng/ml are present in about half, and hypofibrinogenemia and hemophagocytosis in about one-fourth and one-third of HLH patients, respectively. However, elevated sCD25 and impaired NK-cell activity can be found in all patients at initial presentation[50]. Therefore, it is of great importance to find more sensitive parameters for the diagnosis of HLH, such as sCD25 and NK-cell activity, for any diagnostic and therapeutic delay usually has dire consequences for patients with HLH. Hypercytokinemia is the major pathologic feature of HLH that is responsible for most, if not all, of the clinical manifestations. We speculate that the elevation of various cytokines may appear at early stages of HLH. On the other hand, due to the fact that those elevated cytokines can also be found in other hyperinflammatory diseases, such as sepsis/MODS (multiorgan dysfunction syndrome), whether a HLH-related and specific spectrum exists should be determined before such a pattern can be used for diagnostic purposes.

In our previous study, we used the cytometric bead array (CBA) technique to quickly determine the serum Th1 and Th2 cytokines, including IFN-γ, TNF, IL-10, IL-6, IL-4, and IL-2, in 24 children with *de novo* HLH and 87 control children. The results showed that the pattern of significant increase of IFN-γ and IL-10 combined with a slightly increased level of IL-6 was specific for childhood HLH. In patients with bacterial sepsis, a different cytokine pattern was identified, which showed an extremely high level of IL-6 and moderate to high levels of IL-10, whereas IFN-γ was only slightly elevated or just normal[46]. Five patients were diagnosed with HLH according to the Th1/Th2 cytokine pattern 3~13 days earlier than they fulfilled the HLH-2004 diagnostic criteria and got timely treatment. Furthermore, the levels of IFN-γ and IL-10 were correlated to the severity of the disease, and could be used as predictors for survival and disease activity. Since 2007, the test has been carried out for more than 10,000 febrile patients in our institution and the results are consistent with our previous findings (data not shown). Presently, Th1/Th2 cytokine determination is a routine test for patients with HLH in our institution and has been for more than 3 years, with both very high sensitivity and specificity.

A study of 1113 patients with fever in the department of hematology/oncology of our hospital showed exciting results. We analyzed the nonparametric ROC curves using the above cytokines for HLH diagnosis. The AUC (area under curve) for IFN-γ was 0.995 with 95% confidence intervals of 0.992–0.999. IFN-γ level >100 pg/ml had a sensitivity of 94.2% and specificity of 97.3% for a diagnosis of HLH. We then combined the biomarkers and set the following criteria for HLH: IFN-γ ≥100 pg/ml, IL-10 ≥60 pg/ml, and the level of IFN-γ is higher than IL-6. These criteria showed a sensitivity of 90.0% and specificity of 99.7% for the diagnosis of HLH (data not shown). Therefore, we strongly recommend that the test be integrated into the current diagnostic criteria proposed by the International Histiocyte Society in the HLH-2004 protocol.

## DIFFERENTIAL DIAGNOSIS

HLH is not a single entity, but a clinical syndrome that can be encountered in association with various underlying conditions leading to similar characteristic clinical and laboratory situations. Generally, HLH can be classified into two categories according to the underlying etiologies: one is the primary (genetic) and the other is the secondary (acquired) form. Primary HLH is inherited in an autosomal-recessive or X-linked manner and can either be FHL or part of an immunodeficiency syndrome. HLH can also be developed in patients without known genetic defects and is often associated with certain triggers. Most commonly, viral infections have been reported in association with HLH development, which is termed as virus-associated hemophagocytosis syndrome (VAHS). Epstein-Barr virus (EBV) has been recognized as a major instigating infection in Asia[51]. Other viruses that trigger VAHS include the herpes virus group, cytomegalovirus, human immunodeficiency virus, influenza virus, and rubella[52,53]. Other microbial pathogens including *Mycobacterium tuberculosis*, *Mycoplasma pneumoniae*, fungi, and parasites can trigger HLH as well[52,54]. These are designated as infection-associated hemophagocytosis syndrome (IAHS). HLH can frequently occur in patients with autoimmune diseases. The most commonly recognized association of HLH with systemic juvenile rheumatoid arthritis (SJRA) is the macrophage activation syndrome (MAS). The recognition of the fact that HLH occurs in patients with certain malignancies, typically lymphoid malignancies, has led to the term malignancy-associated hemophagocytic syndrome (MAHS) or lymphoma-associated hemophagocytic syndrome (LAHS). Furthermore, HLH may occasionally develop later during cancer treatment as a consequence of treatment-related immunosuppression[55]. As HLH is a very heterogeneous disorder, once a diagnosis of hemophagocytic syndrome is established, a search for an underlying cause is necessary.

The newly expanded International Histiocyte Society guidelines for the diagnosis of HLH assist this population to receive appropriate therapy promptly. However, as the diagnosis is usually made largely relying on the nonspecific clinical features, many patients with severe sepsis, systemic inflammatory response syndrome (SIRS), and MODS may also meet the diagnostic criteria for HLH. Fever, cytopenia of two lines, hypertriglyceridemia, hypofibrinogenemia, and high levels of ferritin are common in patients with sepsis/SIRS/MODS[56]. Hemophagocytosis has also been reported specifically in patients with SIRS and in pediatric patients with MODS[57,58]. Elevated sCD25 and decreased NK activity, although more distinctive for HLH, are also used as predictive markers in sepsis[59,60]. Therefore, many patients with sepsis/SIRS/MODS may meet the current HLH diagnostic criteria and are at risk of receiving chemotherapy, which may suppress their immune system and exacerbate the existing infection or even cause opportunistic infection. Another situation that clinicians should be alert to is recurrent fever in HLH patients who have achieved remission, which may be caused by infections due to complications of immunosuppressive therapy or reactivation of HLH. For the first etiology, specific antimicrobial therapies for pathogens, such as intensive antibiotics, should be given, and chemotherapy may aggravate the infection. For the second causative mechanism, the patient's immune system should be further suppressed; otherwise, the overwhelming active disease may be fatal. Therefore, it is of great importance to differentiate recurrent HLH from infection in order to avoid inappropriate and even contraindicated treatment.

The correlation between HLH and sepsis/SIRS/MODS has not been well illustrated. However, an interesting concept has been proposed by Castillo and Carcillo in which the conditions of sepsis/SIRS/MODS/HLH form a continuum of immune dysregulation in the presence of a trigger. In other words, when clear genetic mutations causing extensive and lethal cytotoxic dysfunction of NK cells and CTLs existed, the patients presented as FHL; while lesser alterations or polymorphisim of genes resulting in partial cytotoxicity defect, SHLH/severe sepsis/MODS can occur under the conditions of the right immune/infectious stimulus[56]. Our results on cytokine profiles determined in HLH and septic patients also seemed to agree with the proposal[46,61]. Younger patients or those with evidences showing genetic defects had higher cytokine levels with more severe symptoms than older patients or those without detectable genetic mutations. This is an important field for investigation as current approaches to the treatment of patients with HLH and severe sepsis are quite different.

Ferritin is a useful and convenient screen in suspected cases of HLH. A review of ferritin levels in pediatric patients found that a ferritin level >10,000 μg/l was 90% sensitive and 96% specific for HLH[62]. Therefore, ferritin has been recommended as a helpful investigational aid for the diagnosis of suspected HLH[63]. Recently, we found that the differentiation of HLH vs. sepsis/SIRS/MODS can be solved by cytokine pattern analysis. Although HLH and severe sepsis are both hyperinflammatory syndromes, they present different cytokine patterns, as discussed above, which may relate to their different pathophysiological processes. Generally, immunosuppressive therapy is necessary in some patients with severe sepsis or septic shock in whom the immune response itself has caused tissue damage[64,65]. However, for the majority of patients, the intensity of this therapy is much less than in HLH, and cyclosporin A (CSA) and etoposide (VP-16) are not recommended for sepsis.

Difficulty in the differential diagnosis of Kawasaki disease (KD) vs. HLH has been frequently reported. The differentiation of these two entities is difficult, as both syndromes are characterized by prolonged fever, and are diagnosed by two different clinical and laboratory scoring systems. Moreover, both KD and HLH are characterized by hypercytokinemia. However, partially different cytokine patterns between the two are identified; IL-6, IL-10, and TNF are elevated in both diseases, and even sCD25 is found to be elevated in KD. However, IFN-γ, a key cytokine for HLH, is within normal range in patients with KD[66,67]. The specific Th1/Th2 cytokine pattern recently identified by our group helps rapid differential diagnosis between these two diseases[68].

## TREATMENT

HLH is a disease that possesses major diagnostic and therapeutic difficulties. In most circumstances, it is a fulminant and rapidly fatal condition. Early diagnosis is very important to a successful outcome. However, HLH may have an insidious course in some patients and some diagnostic criteria often appear late during the course of the disease. In addition, the development of HLH is associated with various underlying conditions. Thus, the treatment of HLH should not be guided only by the severity of the condition, but should also depend on the underlying cause of the disease. The treatment of HLH should include the suppression of the severe hyperinflammation, the killing of pathogen-infected cells, treatment to restore normal organ function, and treatment of underlying diseases, i.e., stem cell transplantation for patients with genetic disorders.

The HLH-2004 protocol comprises phases of initial therapy and continuation therapy. Initial treatment is based on dexamethasone (DXM), CSA, and VP-16, which is designed to control and induce a remission of the symptoms and signs of the disease by inhibiting the activation of lymphocytes and suppressing the hypercytokinemia. Owing to the potential risk of secondary malignancies, VP-16 is not recommended in mild cases. For most patients with SHLH or less severe forms of HLH, the use of corticosteroids and high-dose immunoglobulin may be sufficient. However, such patients should be intensively followed, since some initially mildly affected patients may deteriorate rapidly. For patients with genetic HLH and with severe signs and symptoms, combination therapy of DXM, CSA, and VP-16 is recommended. HLH in immunodeficiency syndromes, such as CHS, GS2, and XLP, also responds to VP-16–containing regimens, but as in FHL, the only potential curative therapy currently available is stem cell transplantation. The clinical course of EBV-associated HLH is often fulminant and the outcome is usually poor. Prompt VP-16 administration (within 4 weeks from diagnosis) can benefit these patients[69]. The treatment of MAHS is difficult to generalize owing to its complex nature, and should be decided based on individual conditions. In 2009, Thompson et al.[69] reported that the up-front use of CSA could increase the risk of central nervous system damage as opposed to the later use of it. This may be caused by the compromised function of the liver, which makes CSA more toxic to the brain, and by hypertension that was also related the high CSA level[70]. However, we still considered that CSA should be administered as early as possible. According to the very high levels of the cytokines we found in patients with HLH at the time of disease onset, a large quantity of cytokines could directly or indirectly damage the brain, liver, and other organs. Early administration of CSA could suppress the cytokine storm at an early stage, and the process to the harmful damage to the brain could be prematurely blocked. In order to reduce the incidence of central nervous system damage, we suggest that the dosage of CSA be reduced in patients with abnormal liver function.

HLH used to be a fatal disease. In 1983, Janka reported a study of 121 patients with an overall 1-year survival of only 5%[71]. After introduction of HLH-94, the estimated 3-year probability of survival rose to 51 ± 20% for FHL and 55 ± 9% for the whole cohort patients[72], a great improvement in the treatment of HLH. The introduction of VP-16 and stem cell transplantation into the protocol was the main reason for the success.

HLH is a disease prone to relapse or to be a chronic, active disease. According to a report based on HLH-94, of the 22% of patients who died during the initial and continuation therapy, four-fifths died from the disease itself[72]. Many patients who were scheduled to receive hematopoietic stem cell transplantation died before disease remission. Unfortunately, few data have been available for the treatment of relapsed or refractory HLH. Antithymocyte globulin (ATG) therapy, which can significantly reduce the number of T lymphocytes, is an efficient treatment for HLH. A study of 38 patients with FHL showed that first-line ATG therapy induced rapid and complete response in 23 (82%) of 28 cases after the first course of treatment, whereas second-line ATG therapy was effective in five (50%) of 10 cases after a first course[73]. There are reports of successful treatment of HLH with the anti-CD25 antibody daclizumab, the anti-CD52 antibody alemtuzumab, and drugs directed at TNF[55,74,75,76]. However, all of these responses are limited to case reports. Recently, a potential future role for targeted immunotherapy has been suggested through the use of mouse models of FHL[77]. Therapeutic administration of an anti–

IFN-γ antibody induced recovery from HLH in both perforin-deficient and RAB27A-deficient mice, as evidenced by increased survival, decreased cytokinemia, and corrections of a series of clinical and histopathological manifestations. Involvement of the central nervous system in RAB27A-deficient mice was prevented by anti–IFN-γ therapy. Although the anti–IFN-γ antibody can only block IFN-γ–mediated macrophage activation, we speculate that CTL activation and expansion may be suppressed indirectly by the decreased secretion of IL-12 and IL-18 by macrophages, which is suggested by the reduced hemophagocytosis in the liver after anti–IFN-γ treatment. Anti–IFN-γ antibody could be an attractive and promising remedy, given that current drugs such as VP-16 or ATG can be very toxic and are far more immunosuppressive than a transient IFN-γ blockade.

## CONCLUSION AND FUTURE DIRECTIONS

Important advances have been made during the last 20 years in the recognition and treatment of HLH. The identification of a variety of genetic abnormalities that lead to the deficiencies of the cytotoxic granules exocytosis pathway has provided a deeper understanding of these disorders. However, these genetic forms only account for 30–70% of FHL cases[6]. Other inherited HLH conditions remain to be molecularly identified, which is essential for the establishment of accurate diagnosis and efficient therapy. In clinical practice, HLH is still often overlooked since the clinical symptoms are also found in immune-competent patients with certain special infections. On the other hand, the current diagnostic criteria are so indistinct that many cases are overdiagnosed and overtreated. Therefore, it can only be hoped that further research advances will identify new biomarkers to revise the criteria and make them more specific, such as we have seen for the Th1/Th2 cytokine pattern, which can effectively distinguish the true HLH from other less severe hyperinflammatory diseases such as sepsis, SIRS, and MODS.

Survival of HLH patients has improved over the past 2 decades from close to 0% to over 60% with the use of cytolytic and immunosuppressive drugs combined with stem cell transplantation. Most deaths occur due to the disease itself; therefore, it is important for us to recognize and treat this condition earlier and physicians should learn to move more rapidly to salvage therapy for nonresponding patients. A considerable challenge remains in the development of more specific therapies with stronger therapeutic activity and less toxicity for achieving remission of the disease.

## ACKNOWLEDGMENTS

We thank Dr. Stuart E. Siegel at the Division of Hematology-oncology, Children's Hospital of Los Angeles, University of Southern California School of Medicine for his critical advice on the manuscript preparation. This work was supported in part by grants from the National Natural and Scientific Fund of China (No. 30971283), the Fund of Zhejiang Provincial Bureau of Science and Technology (No. 2007C23007), and the Zhejiang Provincial Natural and Scientific Fund (No. Z205166).
